# Patient Navigation Needs and Quality of Life Among Women with Gynecological Cancer in Indonesia: A Cross-Sectional Study

**DOI:** 10.3390/healthcare14101388

**Published:** 2026-05-19

**Authors:** Hartiah Haroen, Tuti Pahria, Hana Rizmadewi Agustina, Gatot Nyarumentang Adhipurnawan Winarno, Citra Windani Mambang Sari, Windy Natasya, Jerico Franciscus Pardosi

**Affiliations:** 1 Department of Community Health Nursing, Faculty of Nursing, Universitas Padjadjaran, Bandung 45363,West Java, Indonesia; citra.windani@unpad.ac.id; 2 Department of Medical-Surgical Nursing, Faculty of Nursing, Universitas Padjadjaran, Bandung 45363, West Java, Indonesia; tuti.pahria@unpad.ac.id; 3 Department of Fundamental Nursing, Faculty of Nursing, Universitas Padjadjaran, Bandung 45363, West Java, Indonesia; hana.rizmadewi@unpad.ac.id; 4 Department of Obstetrics and Gynecology, Faculty of Medicine, Universitas Padjadjaran, Hasan Sadikin Hospital, Bandung 45363, West Java, Indonesia; gatotnaw@yahoo.com; 5 Department of Nursing, Hasan Sadikin Hospital, Bandung 45363, West Java, Indonesia; natasyawindy@gmail.com; 6 School of Public Health and Social Work, Faculty of Health, Queensland University of Technology, Brisbane 4000, Australia; jerico.pardosi@qut.edu.au

**Keywords:** cancer, patients navigation, quality of life, supportive care needs

## Abstract

**Background**: Patient navigation has been recognized as a promising strategy to address fragmented cancer care; however, evidence from low- and middle-income countries (LMICs) remains limited, particularly regarding how navigation-related needs are associated with patient-reported outcomes. **Objective**: This study aimed to examine the association between multidimensional patient navigation needs and quality of life (QoL) among women with gynecological cancer in Indonesia. **Methods**: A cross-sectional study was conducted among 128 women diagnosed with gynecological cancer at a referral hospital in Indonesia. Patient navigation needs were assessed using a 37-item multidimensional instrument developed based on international frameworks, while QoL was measured using the EORTC QLQ-C30. Data were analyzed using Pearson correlation and multiple linear regression to evaluate the relationships and relative contributions of navigation need domains to QoL. **Results**: The mean global health status score indicated relatively low QoL (Mean = 41.7, SD = 31.0). Most domains of patient navigation needs were significantly and negatively associated with QoL (*p* < 0.001), with the strongest correlation observed for total navigation needs (r = −0.657). Multivariable analysis showed that administrative and financial needs showed the strongest association with poorer QoL (β = −0.373, *p* < 0.001), followed by psychosocial, cultural, and family support needs (β = −0.356, *p* < 0.001). In contrast, late-stage clinical needs were positively associated with QoL (β = 0.206, *p* = 0.005). The model explained 59.5% of the variance in QoL. **Conclusions**: Patient navigation needs are strongly associated with QoL among women with gynecological cancer, highlighting the critical role of system-level and psychosocial factors in shaping patient outcomes. Addressing administrative complexity, financial burden, and psychosocial support gaps is essential for improving QoL in LMIC settings. These findings provide novel evidence for developing context-specific, integrated patient navigation models to enhance cancer care delivery.

## 1. Introduction

Cancer remains a major global health challenge, with more than 19 million new cases and nearly 10 million deaths reported annually [1,2]. Among women, gynecological cancers including cervical, ovarian, and uterine cancers contribute substantially to the overall disease burden and exert profound effects on quality of life (QoL), encompassing physical, psychological, and social dimensions [3,4]. In Indonesia, cancer is also a leading cause of mortality, with cervical cancer being the most prevalent type among women [5]. The burden is particularly pronounced in low- and middle-income countries (LMICs), where limited access to early detection and treatment services results in delayed diagnosis and poorer outcomes [5,6,7].

The increasing number of gynecological cancer survivors in Indonesia has not been accompanied by adequate provision of supportive care. Emerging evidence suggests that unmet supportive care needs particularly in the domains of information, psychological support, and practical assistance are strongly associated with poorer QoL and increased psychological distress among cancer patients [8,9,10]. These unmet needs are directly associated with poorer quality of life and heightened psychological distress, which may subsequently lead to treatment non-adherence, discontinuation of therapy, and unfavorable health outcomes [10,11].

These challenges are further compounded by the complexity of the healthcare system. The cancer care trajectory involves multiple interconnected stages; however, in practice, it is often constrained by structural limitations such as a tiered referral system, unequal distribution of healthcare resources, and sub-optimal coordination among providers [12,13,14]. In addition, non-clinical barriers including limited health literacy, financial constraints, and geographical obstacles further exacerbate disparities in access to care [7,15]. Collectively, these factors contribute to fragmented care pathways and negatively impact patients’ QoL.

Although the concepts of unmet needs and patient navigation needs are closely related, they represent different conceptual perspectives within cancer care. Unmet needs generally refer to the discrepancy between supportive care needs and the support or services actually received by patients across physical, psychological, informational, social, and practical domains [16,17,18]. In contrast, patient navigation needs specifically focus on the barriers, coordination challenges, and assistance required for patients to effectively access and navigate healthcare systems throughout the cancer care continuum, including referral processes, administrative procedures, financial access, communication with healthcare providers, and continuity of care [15,19]. Therefore, while unmet needs primarily reflect insufficiently addressed patient outcomes, patient navigation needs emphasize healthcare system processes and structural barriers that may contribute to those unmet needs if inadequately managed. This distinction is particularly important in LMIC settings such as Indonesia, where fragmented healthcare systems and limited care coordination may further complicate cancer care delivery.

Patient navigation has been proposed as a strategic intervention to address barriers or challenges by guiding patients through complex healthcare systems and ensuring timely access to appropriate services [15,20]. In high-income countries, patient navigation was demonstrated to be effective in improving care coordination, treatment adherence, and patient-reported outcomes, including QoL [14,21]. However, its application in LMICs remains limited and insufficiently explored, particularly in terms of how navigation-related needs are experienced and how they influence patient outcomes [22].

In Indonesia, the high prevalence of unmet needs further underscores the urgency of more integrated and patient-centered approaches. Approximately 98% of gynecological cancer survivors report at least one unmet need, particularly in relation to information, comprehensive care, and QoL [8]. Moreover, caregivers also experience substantial unmet needs, including financial, legal, and emotional support, which are often overlooked within the current healthcare framework [8,23].

Despite these findings, there is limited research on patient navigation systems tailored to the Indonesian context. Existing studies have focused on validating tools to assess unmet needs [9,24], but there is a lack of actionable frameworks or interventions to address the gaps. The absence of a clear understanding of patient navigation needs may result in interventions that are insufficiently targeted and less effective in improving patient outcomes [21]. This highlights a critical conceptual and empirical gap in the literature, particularly within the Indonesian context.

Several key research gaps can therefore be identified. First, there is a lack of structured and integrated patient navigation systems within the Indonesian healthcare setting [14,25,26]. Second, the high prevalence of unmet needs continues to adversely affect patients’ QoL [8,10]. Third, caregiver needs remain largely neglected within the existing care frameworks [23]. Fourth, there is a paucity of culturally appropriate and context-specific interventions tailored to the Indonesia setting [9,24]. This study goes beyond unmet needs by operationalizing patient navigation needs into a multidimensional construct and empirically testing its predictive role on QoL in a low- and middle-income country (LMIC) context. Accordingly, this study aims to examine the relationship between patient navigation needs and QoL among women with gynecological cancer in Indonesia. The findings are expected to provide empirical evidence for developing contextually relevant, integrated, and patient-centered navigation interventions to improve QoL and overall cancer care delivery.

## 2. Materials and Methods

### 2.1. Study Design

A cross-sectional design was utilized to examine the association between patient navigation needs and QoL among women with gynecological cancer. Data collection was conducted from August to October 2025. This manuscript was drafted in accordance with the Strengthening the Reporting of Observational Studies in Epidemiology (STROBE) guidelines for reporting observational (cross-sectional) research.

### 2.2. Sample and Setting

The study population included women diagnosed with gynecological cancer who were receiving treatment at a major referral hospital in West Java, Indonesia. Participants were selected through a convenience sampling method based on predefined inclusion criteria: (1) a confirmed diagnosis of gynecological cancer, (2) age ≥18 years, and (3) the ability to read and comprehend the study questionnaire. Exclusion criteria comprised patients with cognitive impairments or severe clinical conditions that hindered independent questionnaire completion, as well as those who refused to participate. Data were collected through direct interaction with patients within the hospital setting.

Sample size estimation was conducted using G*Power version 3.1.9.7 to ensure sufficient statistical power. The calculation was based on a linear multiple regression model (fixed model, R^2^ deviation from zero), assuming a medium effect size (f^2^ = 0.15), a significance level of 0.05, and 80% statistical power. With five predictors included in the model, the minimum required sample size was 92 participants (actual power = 0.80). To account for a potential 10% rate of attrition or incomplete responses, the target sample size was increased to at least 102 participants. Ultimately, a total of 128 participants were included in the study, exceeding the minimum requirement.

### 2.3. Data Collection

Data were collected through direct recruitment of patients hospitalized in the gynecologic oncology inpatient wards of a tertiary referral hospital in Indonesia. Eligible participants were identified by the research team based on the predefined inclusion criteria during their hospitalization period. The inclusion criteria were: (1) women diagnosed with gynaecological cancer, (2) aged ≥ 18 years, (3) hospitalised in the gynecologic oncology inpatient ward during the study period, (4) able to communicate verbally and understand the Indonesian language, and (5) physically and psychologically stable at the time of data collection. Patients who experienced clinical deterioration, severe pain, decreased consciousness, cognitive impairment, or any condition that prevented effective communication and questionnaire completion were not included in the study.

Prior to recruitment, the research team coordinated with healthcare professionals in the inpatient wards to facilitate access to potential participants and ensure that patients were in stable condition to participate in the study. Eligible patients were approached individually and provided with a comprehensive explanation regarding the study objectives, procedures, voluntary nature of participation, and confidentiality of the data. Those who agreed to participate were asked to provide written informed consent before completing the study questionnaire. All data collection procedures were conducted within the inpatient ward setting.

### 2.4. Research Instrument

#### 2.4.1. Patient Navigation Needs

Patient navigation needs were assessed using a self-developed questionnaire constructed by the researchers, as no standardized instrument specifically measuring patient navigation needs in the Indonesian context was available. The development of the instrument was informed by a comprehensive review of internationally recognized frameworks and tools, including the Care Coordination and Continuity of Care Questionnaire—Patient version (CCCQ-P/CCCQP) and the Patient Navigation Barriers and Outcomes Tool (PN-BOT), as well as patient navigation guidelines from the World Health Organization (WHO), particularly those addressing navigation across the diagnostic and treatment continuum [20,27,28].

The instrument was designed to capture multidimensional aspects of patient navigation needs, including access to care, coordination of services, information and health literacy, communication with healthcare providers, psycho-social and family support, and clinical and socioeconomic barriers. All items were adapted and contextualized to reflect the Indonesian healthcare system and sociocultural setting.

The instrument consisted of 37 items designed to capture the multidimensional aspects of patient navigation needs. These items were organized into eight domains: (A) Access and Logistics (items 1–6), (B) Administration and Finance (items 7–11), (C) Information, Education, and Health Literacy (items 12–17), (D) Communication and Team Coordination (items 18–22), (E) Psycho-social, Cultural, and Family Support (items 23–27), (F) Early-Stage-Specific Clinical Needs (items 28–30), (G) Late-Stage-Specific Clinical Needs (items 31–34), and (H) Sociolegal and Socioeconomic Barriers (items 35–37).

Each item was developed to reflect common barriers and support needs encountered by gynecological cancer patients within the Indonesian healthcare system, taking into account sociocultural, economic, and health system-related factors. The instrument was designed to comprehensively assess patients’ needs across the entire cancer care trajectory, from early detection to palliative care.

Responses were measured using a five-point Likert scale (0–4) based on patients’ experiences over the past month, where 0 = no need for assistance, 1 = very little need, 2 = low need, 3 = moderate need, and 4 = high need for assistance. Higher scores indicate greater patient navigation needs. Domain scores were calculated as the mean of item scores within each domain, while the total score was computed as the average of all items, with higher scores reflecting greater overall navigation needs.

Given that the instrument was newly developed, psychometric evaluation was conducted prior to its use in the main study. Content validity was established through expert review involving oncology clinicians and nursing academics. Further evaluation using the Rasch model supported these findings. The Rasch analysis indicated that the instrument demonstrated good overall psychometric properties (Table 1). The scale consisted of 37 items and showed excellent internal consistency, with a Cronbach’s alpha of 0.92.

The raw variance explained by the measures was 34.1%, exceeding the minimum acceptable threshold for unidimensionality, although still indicating a moderate strength of the primary construct. The unexplained variance in the first contrast was 3.1, which is slightly above the recommended cutoff of 2.0, suggesting the presence of minor secondary dimensions. However, this value remains within an acceptable range for exploratory instrument development.

In terms of reliability, the person reliability was 0.89 with a separation index of 2.90, indicating that the instrument was able to distinguish respondents into multiple levels of ability with good precision. The item reliability was also high (0.95) with an item separation index of 4.29, suggesting that the sample size was adequate to confirm stable item difficulty estimates and that the items were well distributed along the latent trait continuum. Overall, these findings indicate that the instrument is reliable and has acceptable construct validity, although slight multidimensionality may exist.

The Rasch analysis demonstrated acceptable item fit and reliability; however, the unexplained variance value (3.1) indicated the presence of potential secondary dimensions within the instrument. This finding is not entirely unexpected, as patient navigation needs are conceptually multidimensional and encompass several distinct but interrelated domains, including administrative, psychosocial, informational, and clinical needs. To further examine the dimensional structure, Confirmatory Factor Analysis (CFA) was conducted and supported the hypothesized multidimensional eight-domain model with acceptable model fit indices. Therefore, the instrument was interpreted as representing a multidimensional construct rather than a strictly unidimensional scale.

In addition to content the validation through expert review and Rasch analysis, construct validity of the instrument was further evaluated using Confirmatory Factor Analysis (CFA). The CFA was conducted to examine whether the hypothesized eight-domain structure of the instrument was empirically supported by the data. The proposed model consisted of eight latent constructs representing key dimensions of patient navigation needs: access and logistics, administration and finance, information and health literacy, communication and coordination, psychosocial and family support, early-stage clinical needs, late-stage clinical needs, and sociolegal/socioeconomic barriers.

Model fit was evaluated using multiple fit indices, including the Comparative Fit Index (CFI), Tucker–Lewis Index (TLI), Root Mean Square Error of Approximation (RMSEA), and Standardized Root Mean Square Residual (SRMR). The CFA demonstrated acceptable-to-good model fit (CFI = 0.901; TLI = 0.942; RMSEA = 0.042; SRMR = 0.093), supporting the multidimensional structure of the instrument. All factor loadings were statistically significant (*p* < 0.05) and exceeded the minimum acceptable threshold of 0.40, indicating adequate convergent validity across domains.

Nevertheless, several domains, particularly early-stage clinical needs and sociolegal/socioeconomic barriers, demonstrated relatively lower factor loadings and larger standard errors, suggesting that further refinement and validation may be necessary. These findings are consistent with the Rasch analysis, which indicated slight multidimensionality (unexplained variance = 3.1). Therefore, the instrument should be considered preliminary and requires additional psychometric testing in larger and more diverse populations.

#### 2.4.2. Quality of Life

QoL was measured using the Indonesian version of the European Organization for Research and Treatment of Cancer Quality of Life Questionnaire Core 30 (EORTC QLQ-C30) [29]. The Indonesian adaptation has demonstrated satisfactory validity, with acceptable convergent validity indicated by item–domain correlations greater than 0.40, as well as good reliability, reflected by Cronbach’s alpha coefficients of ≥0.70 across domains [29]. Raw scores for each domain were calculated by averaging the relevant items and were then linearly transformed into a 0–100 scale following the EORTC scoring manual. On this scale, higher scores in the functional and global health status domains represent better functioning and overall quality of life, whereas higher scores in the symptom domains reflect a greater level of symptom burden [29].

### 2.5. Data Analysis

Data were analyzed using statistical software (Jamovi version 2.4.7). Descriptive (univariate) analysis was conducted to summarize participants’ sociodemographic and clinical characteristics, as well as their patient navigation needs and quality of life variables. Continuous data were presented as mean, standard deviation, minimum, and maximum values. Bivariate analysis was performed to examine the relationships between patient navigation needs and quality of life domains. As all variables were treated as continuous, Pearson’s correlation coefficient was applied. Prior to conducting the correlation analysis, normality was assessed using the Kolmogorov–Smirnov test, and all variables satisfied the normality assumption (*p* > 0.05). The analysis included all domains of patient navigation needs and QoL.

Prior to conducting the regression analysis, several assumption tests were performed to ensure the adequacy of the model. Normality of the data was assessed using the Shapiro–Wilk, Kolmogorov–Smirnov, and Anderson–Darling tests, all of which indicated that the data were normally distributed (*p* > 0.05). Heteroscedasticity was evaluated using the Breusch–Pagan, Goldfeld–Quandt, and Harrison–McCabe tests. The Breusch–Pagan test showed a significant result (*p* = 0.004), indicating potential heteroscedasticity; however, both the Goldfeld–Quandt (*p* = 0.833) and Harrison–McCabe tests (*p* = 0.840) suggested that the assumption of homoscedasticity was met. Autocorrelation was examined using the Durbin–Watson test, which indicated no evidence of autocorrelation (DW = 1.89, *p* = 0.496). Multicollinearity was assessed using collinearity statistics. All variance inflation factor (VIF) values were below 10 (range: 1.30–3.16), and tolerance values ranged from 0.317 to 0.772, indicating no multicollinearity among the independent variables. Standardized regression coefficients were used to determine the most influential predictors in the final model. A *p*-value of <0.05 was considered statistically significant for all analyses.

### 2.6. Ethical Consideration

This study received ethical approval from the Universitas Padjadjaran Research Ethics Committee (Approval No. 709/UN6.KEP/EC/2025) on 19 August 2025. The approval indicates that the study was conducted in accordance with internationally recognized ethical standards for research involving human participants. All procedures adhered to the principles of the Declaration of Helsinki, ensuring respect for human dignity, protection of participants’ rights and well-being, and fair treatment throughout the research process.

Prior to participation, all respondents were provided with comprehensive information regarding the study objectives, procedures, as well as potential benefits and risks. Written informed consent was obtained from each participant before data collection began. Participation was entirely voluntary, and no form of coercion was involved. Confidentiality and anonymity were strictly maintained by removing all personally identifiable information from the data set. All data collected were used exclusively for research purposes and were securely stored to safeguard participants’ privacy.

## 3. Results

### 3.1. Characteristics of Participants

Based on Table 2, the majority of respondents were aged 41–60 years, accounting for 77 individuals (60.2%). Most respondents were married (93 individuals; 72.7%), and nearly all identified as Muslim (126 individuals; 98.4%). The largest proportion of respondents were of Sundanese ethnicity (94 individuals; 73.4%), and most were unemployed (104 individuals; 81.3%). In terms of income, the majority earned <60 USD (80 individuals; 62.5%), and all respondents (100%) had health insurance coverage. Regarding cancer type, most respondents were diagnosed with cervical cancer (108 individuals; 84.4%), with Stage III being the most common stage (38 individuals; 29.7%). Additionally, the majority had experienced illness for less than one year (106 individuals; 82.8%).

### 3.2. Patient Navigation Needs Based on Mean Rank

Based on the results of patient navigation needs (Table 3), the needs with the highest mean values were nutritional support and wound/perineum care with a mean of 3.34 (SD = 0.958). Meanwhile, the need with the lowest mean was the need for follow-up after a visit/treatment (via telephone/chat) with a mean of 3.09 (SD = 1.111).

### 3.3. Patient Navigation Needs and Quality of Life

Based on Table 4, for Patient Navigation Needs, the domain with the highest mean score was Information, Education & Health Literacy (Mean = 18.36, SD = 5.97), followed by Communication and Team Coordination (Mean = 15.35, SD = 5.09) and Psychosocial, Cultural & Family Support (Mean = 14.90, SD = 4.73). In contrast, the lowest mean scores were found in Early-Stage-Specific Clinical Needs (Mean = 6.34, SD = 2.83) and Sociolegal and Socioeconomic Barriers (Mean = 6.45, SD = 2.87). Overall, the total patient navigation needs score had a mean of 101.13 (SD = 23.50), with a minimum score of 12 and a maximum score of 141.

Regarding QoL, within the functional domain, the highest mean score was observed in Cognitive Functioning (Mean = 71.4, SD = 32.3) and Social Functioning (Mean = 68.0, SD = 30.3), whereas the lowest mean score was found in Role Functioning (Mean = 43.1, SD = 35.7). In the symptom domain, the highest mean scores were reported for Diarrhea (Mean = 89.3, SD = 26.4) and Dyspnea (Mean = 80.5, SD = 31.7), while the lowest mean score was observed for Fatigue (Mean = 31.7, SD = 28.8). Overall, the Global Health Status score showed a mean of 41.7 (SD = 31.0), with scores ranging from 0 to 100.

**Figure 1:**
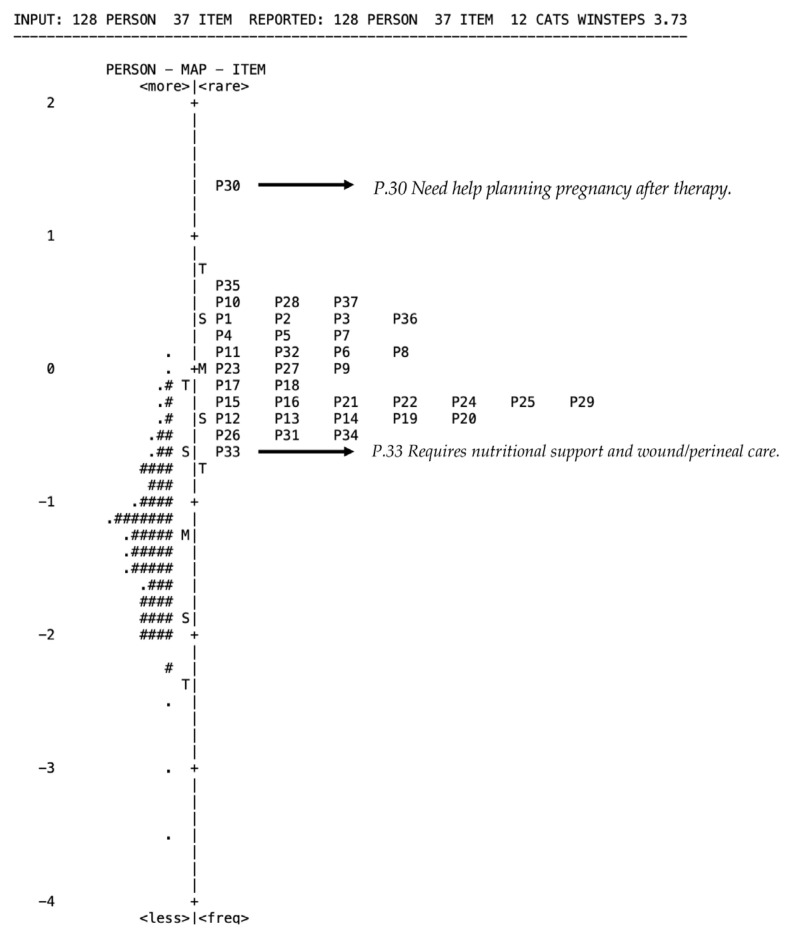
Wright Map of Patients’ Navigation. Each # represents a group of respondents at a particular logit position, while a. indicates a smaller number of respondents at that level. The greater the number of # symbols, the higher the concentration of respondents with similar levels of need.

Most respondents are clustered between 0 and −2 logits, indicating that the majority of women with gynecological cancer in this study experience moderate to relatively high levels of patient navigation needs (Figure 1). Meanwhile, most items are located around the 0 logit level, suggesting that the instrument items are well targeted to the respondents’ level of need and are able to measure patient navigation needs appropriately. The most difficult item is P30 (Need help planning pregnancy after therapy), positioned at the highest logit level. This indicates that post-treatment pregnancy planning is the least commonly reported or most specific need among respondents. In contrast, P33 (Requires nutritional support and wound/perineal care) is located at a lower logit level, showing that this need is more common and widely experienced by patients.

### 3.4. Correlation Between Patient Navigation Needs and QoL

**Figure 2:**
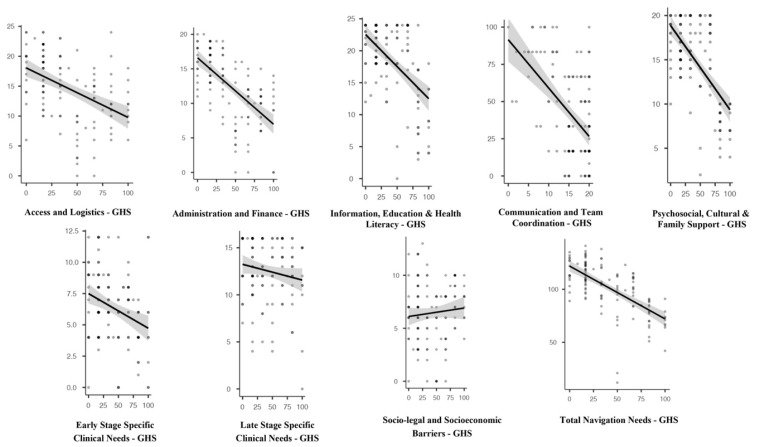
Correlation between Patient Navigation Needs and Global health status.

Based on Table 5 and Figure 2, Pearson correlation analysis showed that most domains of patient navigation needs were significantly and negatively correlated with global health status (QoL). Significant negative correlations were found for access and logistics (r = −0.454, *p* < 0.001), administration and financing (r = −0.621, *p* < 0.001), information, education & health literacy (r = −0.521, *p* < 0.001), communication and team coordination (r = −0.533, *p* < 0.001), psychosocial, cultural & family support (r = −0.623, *p* < 0.001), and early-stage-specific clinical needs (r = −0.303, *p* < 0.001). The strongest negative association was observed in the total navigation needs score (r = −0.657, *p* < 0.001), indicating that higher overall navigation needs were associated with poorer global health status/QoL. In contrast, late-stage-specific clinical needs (r = −0.154, *p* = 0.084) and sociolegal and socioeconomic barriers (r = −0.086, *p* = 0.333) were not significantly correlated with global health status/QoL.

### 3.5. Association Between Patient Navigation Needs on Global Health Status

The multivariable linear regression analysis (Table 6) demonstrated that patient navigation needs significantly influenced global health status (quality of life) among gynecological cancer patients, with the model explaining 59.5% of the variance (Adjusted R^2^ = 0.595). This indicates that the included domains of patient navigation needs collectively have a substantial contribution to patients’ QoL.

Among the predictors, administration and finance emerged as a significant negative predictor of global health status (β = −0.373, *p* < 0.001). Higher scores in this domain reflected greater unmet administrative and financial navigation needs, indicating that patients experiencing higher levels of unmet needs tended to report poorer quality of life. Similarly, psychosocial, cultural, and family support needs were significantly and negatively associated with global health status (β = −0.356, *p* < 0.001), suggesting that greater unmet supportive care needs in these domains were associated with lower perceived QoL.

In contrast, late-stage-specific clinical needs showed a significant positive association with global health status (β = 0.206, *p* = 0.005). In this study, higher scores in the late-stage clinical needs domain reflected greater perceived unmet navigation and supportive care needs. This finding indicates that participants reporting higher late-stage clinical needs also reported higher global health status scores. Given the cross-sectional nature of the study and the counterintuitive direction of the association, the findings should be interpreted cautiously and explored further in future studies.

## 4. Discussion

This study demonstrates that patient navigation needs are significantly associated with QoL among women with gynecological cancer, highlighting the critical role of health care system navigation in shaping patient outcomes. The relatively low global health status observed in this study underscores the substantial burden experienced by patients and suggests that clinical care alone is insufficient to address the multidimensional challenges faced during the cancer trajectory. The relatively low average global health status value (Mean = 41.7) indicates that most patients still experience disabilities in various aspects of life. This finding is similar to a previous study in Indonesia that reported low QoL in gynecological cancer patients due to high unmet needs [8,23]. In addition, research in Asian countries shows that the most frequently unmet supportive care needs include the physical and activities of daily living, psychological, and health care systems and information domains [30,31] These unmet needs are associated with a lower QoL [31,32].

The findings indicate a high demand for patient navigation needs, particularly in the domains of information, education, and health literacy. The most prominent needs included clinical support (nutrition, wound/perineal care, and symptom management), treatment-related information, as well as psychosocial and spiritual support, reflecting the complex and multidimensional challenges experienced by women with gynecological cancer (Table 3). The Wright map analysis further showed that most respondents were located below the mean item difficulty, suggesting that several navigation needs were relatively difficult to recognize or endorse. This finding may indicate limited patient awareness and health literacy regarding available support services, as well as a potential mismatch between patient capacity and the complexity of healthcare navigation demands. Similar findings have been reported in previous Indonesian and Asian studies, where informational, psychological, and healthcare system-related unmet needs were associated with poorer QoL among cancer patients [8,33,34,35,36,37]. Low health literacy may further impair clinical decision-making, increase anxiety, reduce treatment adherence, and limit patients’ ability to access appropriate support services, ultimately affecting QoL [33,34,35,36,37].

A key finding of this study is that patient navigation needs particularly in administrative, financial, and psychosocial domains are strongly associated with poorer QoL (β = −0.373, *p* < 0.001). This finding is highly relevant to the Indonesian context, where, although most patients have health insurance, complex administrative processes, indirect costs (transportation, accommodation), and uncertainty about financing still pose a significant burden for patients and their families [37]. Previous studies have also shown that financial difficulties are correlated with QoL in cancer patients, especially in LMICs [38,39,40,41,42]. In Southeast Asia, patients who were already living in economic hardship before diagnosis reported a significantly lower QoL after developing cancer [43].

Psychosocial, cultural, and family support also emerged as a significant variable associated with QoL (β = −0.356, *p* < 0.001), reinforcing the importance of social context in cancer care. In collectivist societies such as Indonesia, family plays a central role in care giving and decision-making processes [8,23]. Support from family, friends, and significant others is correlated with higher physical, emotional, social, and environmental quality of life in gynecologic cancer patients [44,45]. In the Asian cultural context (Taiwan, China, Indonesia), family and partner support is emphasized as very important because of strong family values [45,46,47,48]. Lack of support leads to loneliness, loss of hope, and more severe psychological symptoms [45].

An interesting finding in this study was the positive association between late-stage-specific clinical needs and QoL (β = 0.206, *p* = 0.005) (Table 6). This finding appears counterintuitive, as advanced clinical needs are generally expected to be associated with poorer quality of life. Several possible explanations may account for this result. Patients with advanced disease may receive more intensive medical attention, including more comprehensive symptom management and supportive care, which could partially contribute to better perceived QoL despite higher clinical needs. In addition, patients with advanced cancer often utilize adaptive coping strategies, such as acceptance, seeking emotional support, positive thinking, and religiosity, whereas maladaptive responses such as denial and emotional suppression tend to be less frequently reported [48,49,50]. Previous studies have also suggested that patients in advanced stages of illness may undergo psychological adaptation as they gradually reconcile their condition with personal goals and expectations [51,52]. This adaptation process may be influenced by coping strategies, emotional regulation, and social support [50,51,52,53].

Nevertheless, these interpretations should be approached cautiously. Alternative explanations, including survivor bias, reverse causality related to the cross-sectional design, and potential measurement-related issues, may also have contributed to the observed association. For example, patients with relatively better functioning may have been more likely to participate in the study or more capable of identifying and reporting their clinical needs. Therefore, the present findings should not be interpreted as evidence that advanced clinical needs improve QoL. Rather, they highlight the complex and potentially non-linear relationship between clinical needs and QoL, which warrants further investigation using longitudinal and psychometric studies.

Importantly, this study also contributes to the theoretical literature by extending the existing literature on unmet supportive care needs through the concept of patient navigation needs. While unmet needs research has focused on identifying deficiencies in supportive care outcomes such as psychological support, information, symptom management, or practical assistance [16,17], the concept of patient navigation needs emphasises the underlying processes and structural barriers that hinder patients from effectively accessing and utilising healthcare services across the cancer care continuum [21,54]. In this study, navigation needs were operationalised as multidimensional barriers related not only to supportive care deficits but also to care coordination, administrative complexity, financial access, communication pathways, continuity of care, and healthcare system navigation. Therefore, the proposed construct offers a more system- and process-oriented perspective than conventional unmet needs frameworks, which primarily capture the discrepancy between patient expectations and received care.

Overall, the regression model in this study was able to explain 59.5% of the variation in QoL, indicating that patient navigation needs are a strong associated with quality of life in women with gynecological cancer patients. However, other factors outside the model also contribute to QoL, such as clinical conditions, social support, and individual psychological factors [47,53,55].

### 4.1. Implications for Practice

The findings also have important implications for practice and policy. First, they underscore the need for structured patient navigation programs that go beyond clinical guidance to include administrative assistance, financial counseling, and psychosocial support. Second, integrating patient navigators into multidisciplinary teams may improve care coordination and reduce fragmentation. Third, the development of context-specific navigation models is essential to address the unique challenges faced in LMIC settings.

### 4.2. Strengths and Limitations

This study has several notable strengths. First, it is among the first to examine patient navigation needs as a multidimensional construct and its association with quality of life among gynecological cancer patients in Indonesia. Second, the use of a contextually developed instrument grounded in international frameworks enhances the relevance and validity of the measurement. Third, the application of multivariable analysis enabled the identification of dominant predictors, with the model explaining a substantial proportion of variance in quality of life (Adjusted R^2^ = 0.595).

However, several limitations should also be considered when interpreting the findings of this study. First, the use of convenience sampling from a single referral hospital may limit the external validity and generalizability of the results. Second, the study population was predominantly composed of women with cervical cancer (84.4%) and patients with stage III or advanced disease, which may introduce selection bias toward individuals with more complex clinical conditions and higher supportive care needs. Therefore, the findings may not fully represent the experiences of patients with other gynaecological cancer types or those in earlier stages of disease. Future multicenter studies involving more diverse cancer types and clinical stages are needed to improve the representativeness and generalizability of the findings. Then, although the instrument demonstrated acceptable preliminary psychometric properties through Rasch analysis and CFA, external validation in broader and more heterogeneous populations has not yet been conducted. Therefore, the findings related to patient navigation needs should be interpreted with caution, and further psychometric refinement is recommended.

Another limitation that should be considered is the unexpected positive association between late-stage clinical needs and QoL. Given the cross-sectional nature of the study, the directionality of this relationship cannot be determined, and reverse causality remains possible. In addition, survivor bias may have influenced the findings, as patients with relatively better functional status and adaptation may have been more likely to participate in the study. Potential measurement-related issues related to the newly developed navigation needs instrument may also have contributed to the observed association. Therefore, this finding should be interpreted cautiously and requires confirmation through longitudinal studies and further psychometric validation.

## 5. Conclusions

This study demonstrates that patient navigation needs are significantly associated with QoL among women with gynecological cancer receiving care at a referral hospital in Indonesia, particularly among patients with predominantly cervical cancer and advanced-stage disease. Several domains were found to be associated with QoL, particularly access and logistics, administration and finance, information and health literacy, communication and coordination, as well as psychosocial and family support. Among these factors, administration and finance emerged as the most dominant predictors, followed by psychosocial, cultural, and family support, indicating that challenges related to healthcare access, financial burden, and insufficient psychosocial support are key associated factors of patients’ QoL. These findings highlight that improving QoL requires not only clinical management but also a comprehensive, patient-centered navigation approach that addresses administrative complexity, financial constraints, gaps in information, and psychosocial support needs. The development of structured and context-specific patient navigation models is therefore essential to enhance care coordination and improve patient outcomes in Indonesia.

## Figures and Tables

**Table 1:** Psychometric Attributes of the Instrument.

Psychometric Attribute	Results
No of items	37
Raw explain variance by measure	34.1%
Unexplained Variance	3.1
Cronbach’s alpha	0.92
Person reliability	0.89
Person separation	2.90
Item reliability	0.95
Item separation	4.29

**Table 2:** Respondents’ Characteristics (n = 128).

Characteristics		Frequency	Percentage
Age	18–40 years old	33	25.8
	41–60 years old	77	60.2
	>60 years old	18	14.1
Marital status	Divorce	29	22.7
	Married	93	72.7
	Single	6	4.7
Religion	Islam	126	98.4
	Christianity	2	1.6
Ethnicity	Batak	1	0.8
	Javanese	33	25.8
	Sundanese	94	73.4
Employment Status	Employed	24	18.8
	Not Employed	104	81.3
Income	USD > 60	48	37.5
	USD < 60	80	62.5
Health Insurance	Yes	128	100
Types of cancer	Ovary	15	11.7
	Uterus	5	3.9
	Cervix	108	84.4
Cancer Stage	Unspecified	31	24.2
	I	22	17.2
	II	30	23.4
	III	38	29.7
	IV	7	5.5
Duration of Illness	<1 Year	106	82.8
	1–3 Years	19	14.8
	>3 Years	3	2.4

**Table 3:** Patient Navigation Needs Based on Mean Rank.

Item	Mean	SD
10 Navigation Needs with the Highest Mean Scores (n = 37)		
1. Nutritional support and wound/perineal care.	3.34	0.958
2. Advanced care planning.	3.29	0.906
3. Spiritual support according to beliefs.	3.26	0.941
4. Symptom management (pain, bleeding, fistula, odor, fatigue).	3.24	1.078
5. Needs a written, easy-to-understand summary of the treatment plan.	3.16	1.078
6. Information on treatment options (surgery, radiation, chemotherapy, combinations).	3.14	1.195
7. A responsive primary contact (navigator).	3.13	1.057
8. Information on side effects and how to manage them.	3.12	1.134
9. An explanation of the diagnosis (precancerous vs. cancerous lesions, stage).	3.11	1.138
10. Follow-up after the visit/treatment (phone/chat).	3.09	1.111

**Table 4:** Patient Navigation Needs and Quality of Life (n = 128).

Variable	Mean	SD	Min	Max
Patient Navigation Needs				
Access and Logistics	14.59	5.65	0	24
Administration and Finance	12.59	4.81	0	20
Information, Education & Health Literacy	18.36	5.97	0	24
Communication and Team Coordination	15.35	5.09	0	20
Psychosocial, Cultural & Family Support	14.90	4.73	2	20
Early-Stage-Specific Clinical Needs	6.34	2.83	0	12
Late-Stage-Specific Clinical Needs	12.55	3.38	0	16
Sociolegal and Socioeconomic Barriers	6.45	2.87	0	13
Total Patient Navigation	101.13	23.50	12	141
Quality of Life				
Functional Domain				
Physical Functioning	54.2	31.3	0	100
Role Functioning	43.1	35.7	0	100
Emotional Functioning	55.4	26.4	0	100
Cognitive Functioning	71.4	32.3	0	100
Social Functioning	68.0	30.3	0	100
Symptom Domain				
Fatigue	31.7	28.8	0	100
Nausea and Vomiting	72.8	32.3	0	100
Pain	32.3	34.1	0	100
Dyspnea	80.5	31.7	0	100
Sleep Disturbance/Insomnia	45.3	40.7	0	100
Loss of appetite	47.7	38.5	0	100
Constipation	72.4	37.0	0	100
Diarrhea	89.3	26.4	0	100
Financial difficulties	42.2	38.2	0	100
Overall QoL				
Global Health Status/QoL	41.7	31.0	0	100

**Table 5:** Correlation between Patient Navigation Needs and Global health status (QoL).

Patient Navigation Needs	Global Health Status		
	*p*-Value	r	Interpretation
Access and Logistics	<0.001	−0.454	Moderate
Administration and Finance	<0.001	−0.621	Moderate
Information, Education & Health Literacy	<0.001	−0.521	Moderate
Communication and Team Coordination	<0.001	−0.533	Moderate
Psychosocial, Cultural & Family Support	<0.001	−0.623	Moderate
Early-Stage-Specific Clinical Needs	<0.001	−0.303	Moderate
Late-Stage-Specific Clinical Needs	0.084	−0.154	NS
Sociolegal and Socioeconomic Barriers	0.333	−0.086	NS
Total Navigation Needs	<0.001	−0.657	Moderate

Note: Pearson Correlation.

**Table 6:** Multivariable Analysis of Factors Associated with Global Health Status.

Predictors	*p*	Stand. Estimates (β)	95% (CI)		Adj. R^2^
			Lower	Upper	
Intercept	<0.001				
Access and Logistics	0.219	−0.093	−0.241	0.055	0.595
Administration and Finance	<0.001 **	−0.373	−0.534	−0.213	
Information, Education & Health Literacy	0.191	−0.127	−0.318	0.064	
Communication and Team Coordination	0.527	−0.061	−0.252	0.129	
Psychosocial, Cultural & Family Support	<0.001 **	−0.356	−0.561	−0.150	
Early-Stage-Specific Clinical Needs	0.238	−0.078	−0.210	0.052	
Late-Stage Specific Clinical Needs	0.005 *	0.206	0.064	0.347	
Sociolegal and Socioeconomic Barriers	0.150	0.087	−0.031	0.206	

Note: * *p* < 0.01; ** *p* < 0.001.

## Data Availability

The data supporting the findings of this study are not publicly available due to ethical considerations and participant privacy protection.
